# BMI1 Is Expressed in Canine Osteosarcoma and Contributes to Cell Growth and Chemotherapy Resistance

**DOI:** 10.1371/journal.pone.0131006

**Published:** 2015-06-25

**Authors:** Mehdi Hayat Shahi, Daniel York, Regina Gandour-Edwards, Sita S. Withers, Roseline Holt, Robert B. Rebhun

**Affiliations:** 1 The Comparative Oncology Laboratory and Center for Companion Animal Health, School of Veterinary Medicine, University of California Davis, Davis, CA, 95616, United States of America; 2 The Department of Pathology, Comprehensive Cancer Center, University of California Davis, Davis, California, United States of America; Hormel Institute, University of Minnesota, UNITED STATES

## Abstract

BMI1, a stem cell factor and member of the polycomb group of genes, has been shown to contribute to growth and chemoresistance of several human malignancies including primary osteosarcoma (OSA). Naturally occurring OSA in the dog represents a large animal model of human OSA, however the potential role of BMI1 in canine primary and metastatic OSA has not been examined. Immunohistochemical staining of canine primary and metastatic OSA tumors revealed strong nuclear expression of BMI1. An identical staining pattern was found in both primary and metastatic human OSA tissues. Canine OSA cell lines (Abrams, Moresco, and D17) expressed high levels of BMI1 compared with canine osteoblasts and knockdown or inhibition of BMI1 by siRNA or by small molecule BMI1-inhibitor PTC-209 demonstrated a role for BMI1 in canine OSA cell growth and resistance to carboplatin and doxorubicin chemotherapy. These findings suggest that inhibition of BMI1 in primary or metastatic OSA may improve response to chemotherapy and that the dog may serve as a large animal model to evaluate such therapy.

## Introduction

Human osteosarcoma is a highly aggressive primary tumor of bone. Advances in cytotoxic chemotherapy and high-dose protocols led to dramatic improvements in survival rates nearly three decades ago. Unfortunately, the prognosis for patients with metastatic disease remains poor, and despite intensive ongoing research, there have been few improvements over the past 30 years. While novel therapeutics continue to be developed and explored, identification and targeting of therapy-resistant tumor cells remains a rational approach to improve current standard of care.

The polycomb group (PcG) proteins have been shown to play a critical role in the development and progression of cancer [1] and additionally play an important role in response to DNA damage [2]. PcG proteins are comprised of two multimeric protein complexes, the polycomb repressive complex 1 (PRC1) and the polycomb repressive complex 2 (PRC2). The B cell-specific Moloney murine leukemia virus integration site 1 (BMI1) is a member of the PRC1 complex of transcriptional regulators and initially recognized as an oncogenic partner of c-Myc in lymphomagenesis [3]. BMI1 is crucial for blood-cell development and the self-renewable potential of a variety of both normal and cancer stem cells [4], and may also play a role in cancer progression through regulation of both p16/INK4a and p14/ARF [5, 6].

Recently, others have identified a small molecule inhibitor of BMI1 (PTC-209) that demonstrates differential cytotoxicity against human colorectal cancer cells while having minimal cytotoxic effects on human peripheral blood mononuclear cells and hematopoietic stem cells at similar concentrations [7]. In addition, PTC-209 exhibited an irreversible decrease in the sphere formation ability of primary colorectal tumors *in vitro* and tumor formation *in vivo* following a limited dilution assay, suggesting that inhibition of BMI1 has the potential to target chemo-resistant cancer-initiating cells.

Recent studies have highlighted a potential role for BMI1 in human OSA growth, migration, and drug resistance, although the individual contribution of BMI1 on the growth of human OSA cells has been inconsistent [8–10]. Nevertheless, BMI1 protein expression was previously reported in 18 out of 32 human OSA samples and expression of BMI1 was subsequently found to contribute to increased cell viability, colony formation, and chemoresistance of human OSA cells *in vitro* [8]. Furthermore, BMI1 significantly contributed to growth of human OSA in a xenograft murine model indicating that targeting of BMI1 may prove clinically useful. However, to the authors’ knowledge, expression of BMI1 in metastatic OSA has not been examined.

Spontaneously arising OSA in the dog represents a powerful model to study the biology and treatment of human OSA and provides several advantages over mouse models [11–13]. We set out to determine whether BMI1 was expressed in canine OSA tissues including a subset of patient-matched primary and metastatic tumors. We observed strong nuclear staining of BMI1 in both primary and metastatic canine OSA. Additional staining of primary and metastatic human OSA tissues demonstrated an identical staining pattern. We further examined expression of BMI1 in multiple canine OSA cell lines and found that inhibition of BMI1 significantly reduced the viability, colony formation, and chemoresistance of canine OSA cells *in vitro*.

## Methods

### Immunohistochemistry

Immunohistochemical staining was performed by the UC Davis Comprehensive Cancer Center shared resources biorepository laboratory. This study was compliant with federal, State, and UC Davis regulations (IRB ID #293828–3) as well as ISBER guidelines (International Society for Biological and Environmental Repositories). Briefly, paraffin sections were deparaffinized and utilizing standard protocols, stained on an automated DAKO platform. A rabbit monoclonal antibody for BMI1 (6964S Cell Signaling) at 1:200 dilution was applied following by an anti-rabbit secondary antibody and DAB chromagen. A board certified Anatomic Pathologist (RGE) examined the sections and scored by the percentage of nuclear positive cells.

### Cell lines

Three canine OSA cell lines (Abrams, D17, and Moresco) and three human OSA cell lines (U2OS, SAOS-2, and LM7) were used in this study. The Abrams and Moresco cell lines were a gift from Dr. Douglas Thamm and Colorado State University [14–17]. The D17 cell line is derived from an osteosarcoma lung metastasis and was purchased from ATCC (Manassas, VA, Cat# CCL-183) [16, 17]. The U20S, SAOS-2, and LM7 cell lines were obtained from the MD Anderson Characterized Cell Line Core Facility [18, 19]. All cell lines were grown in Dulbecco’s Modified Eagle Medium (DMEM) supplemented with 10% Fetal Bovine Serum (FBS) and 1x penicillin/streptomycin, all from Life Technologies (Invitrogen), and incubated at 37°C in 5% humidified CO_2_. Canine osteoblast cells (Cn406-05) were purchased from Cell Application Inc.

### Drugs

Doxorubicin (Dox, 2mg/mL, #445–9202) and Carboplatin (Carbo, 10mg/mL, #356–6551) was supplied by Cardinal Health and purchased through the UCDavis VMTH Pharmacy. PTC-209 (#SML1143) was purchased from Sigma-Aldrich and dissolved in DMSO for a stock concentration of 10mM and stored at -80°C.

### BMI1 Transfection with siRNAs

Custom designed Canine BMI1 siRNAs, siRNA-1 and siRNA-2, were made by Life Technologies and the sequences used for each were as follows: BMI1 siRNA-1 (Catalogue #4399665 ID # s453495) Sense: 5’ GACCUAAAUUUGUACAGUATT 3’, Antisense: 5’ UACUGUACAAAUUUAGGUCAA 3’, and BMI1 siRNA 2 (Catalogue #4399665 ID #s453496) Sense:5’ GAAAAUAGCUAAGACUUUATT 3’, Antisense: 5’ UAAAGUCUUAGCUAUUUUCTA 3’. Silencer Negative Control No. 1 siRNA was purchased from Life Technologies. All siRNAs were reconstituted in sterile water for a stock concentration of 50μM. To first determine the knockdown efficiency of each BMI1 siRNA, 70,000 Abrams cells were seeded into each well of 6-well plates with 10% FBS media and allowed to adhere. The media was then changed to DMEM with 2% FBS and cells were incubated for 24hrs. Treatment specific transfection solutions were made by combining siRNA with Lipofectamine and further diluted using Opti-MEM I reduced serum media (Invitrogen). 100nmols of BMI1 or control siRNAs was added to each well and cells were transfected for 24hrs. After transfection, the media was replaced with fresh 2% FBS media and cells were incubated for an additional 72hrs prior to RNA and protein extraction.

### Protein extraction and western blot

Plated cells were washed three times with ice-cold PBS and then lysed with RIPA buffer (Thermo-Pierce). Protein lysates were clarified with centrifugation and total protein was quantified using a BCA Protein Assay (Thermo-Pierce). 20–30μg of protein was then separated using polyacrylamide gel electrophoresis (PAGE) and transferred to a nitrocellulose membrane. Membranes were blocked with 5% milk in PBST and incubated with primary antibodies overnight at 4°C. The primary antibodies used were rabbit anti-BMI1 (1:2000, Cell Signaling #6964S), mouse anti-p16 (1:800, BD Biosciences #51-1325GR) mouse anti-actin (1:20,000, Santa Cruz #56459), and rabbit anti-GAPDH (1:3000, Santa Cruz #25778). Membranes were then washed and incubated with an HRP-conjugated anti-rabbit (Thermo-Pierce Cat# 31464) or anti-mouse (Thermo-Pierce Cat# 31450) secondary antibody for 2hrs, washed again, then visualized with SuperSignal West Femto Maximum Sensitivity Substrate (Thermo #34095). Images were captured and exposure was optimized using Protein Simple FluorChemE (Bio-Techne, San Jose, CA). Figures were arranged using Adobe Photoshop software (version 11.0.2). Quantification was performed using ImageJ 1.48v software (NIH, http://imagej.nih.gov/ij).

### Real Time RT PCR (qRT-PCR)

RNA was extracted from cell lines using the RNAeasy Plus Mini Kit (Qiagen #74134) and 2μg of total RNA was converted to cDNA using the High Capacity RNA-to-cDNA Kit (Applied Biosystems #4387406). BMI1 expression was measured with the TaqMan Dog BMI1 expression assay (Applied Biosystems Assay #Cf02663120_m1) using the StepOne Plus Real-Time RT PCR system (Applied Biosystem). BMI1 expression was normalized to HPRT1 (Applied Biosystems Assay # Cf02626256_m1) and analysis was performed using the delta delta Ct (ΔΔCt) method. Statistical analysis was performed using Kruskal-Wallis with Dunn’s multiple comparisons test.

### MTS/MTT cell proliferation assays

MTS assays were used to assess proliferation of Abrams canine OSA cells following BMI1 siRNA-mediated silencing. Transfection with BMI1 and negative control siRNAs were carried out similarly as described above. Abrams cells were seeded in 96-well plates (7k cells/well), allowed to adhere, then switched to DMEM media with 2% FBS for 24hrs. Cells were then transfected for 24hrs with negative control and BMI1 siRNA-1 at a concentration of 10nmols per well. Cell viability was analyzed at 24hr and 48hrs post transfection using an MTS assay. Briefly, all media was removed and fresh media (DMEM 10%FBS) containing MTS reagent [3-(4,5-dimethylthiazol-2-yl)-5-(3-carboxymethoxyphenyl)-2-(4-sulfophenyl)-2H-tetrazolium] was added to each well and incubated for 2hrs. Absorbance at 490nm was measured with a spectrophotometer (Spectramax 190, Molecular Devices LLC). Each group contained 6 wells and mean absorbance is expressed as a percentage of untreated control cells. Statistical analysis was performed using student’s two-tailed t-test.

MTT assays were used to assess proliferation of Abrams, D17, and Moresco canine OSA cells following treatment of PTC-209 alone and in combination with Dox or Carbo. 500 cells were seeded in 96 well plates with DMEM/10%FBS and allowed to adhere overnight (16–18hrs). For single treatment PTC-209 experiments, cells were incubated with drug for 72hrs at final concentrations of 0, 200, 300, 400, 500, and 600nM. For combination treatment experiments, cells were incubated with drug(s) for 72hrs at the following final concentrations: PTC-209 (0, 100, 200, and 500nM), Dox (0, 3, and 30nM), Carbo (0, 3, and 30μM). Vehicle controls included DMSO (PTC-209), 0.9% saline (Dox), and water (Carbo). Additional controls included untreated (UT) cells (no veh or drug) and wells containing media (DMEM/10%FBS) alone (to assess background absorbance). Briefly, MTT solution was added to each well at a final conc. of 0.5mg/mL and incubated at 37°C for 4hrs. 200uL of DMSO was added to dissolve formazin crystals and absorbance was measured at 570nM and 630nM (reference wavelength) using a spectrophotometer (Spectramax 190, Molecular Devices LLC). 6 wells per group were used for PTC-209 single treatment experiments, and 4 wells per group were used for combination treatment experiments, and all experiments were repeated twice. Statistical analysis was performed using 2-way ANOVA with Tukey’s multiple comparisons test.

### Clonogenic assay

For clonogenic assays involving BMI1 siRNA meditated silencing, 500 Abrams cells were seeded in 6-well plates with DMEM media containing 10% FBS for 24hrs. The media was then changed to DMEM with 2% FBS and transfection solutions containing 15nmols of negative control or BMI1 siRNA-1 were added to each well. After 24hrs of transfection, the media was replaced with fresh 2% FBS media and cells were immediately treated with Carbo at 0, 0.5, or 0.75μM final concentrations, or Dox at 0, 2.5, or 5.0nM final concentrations. Cells were incubated with drug treatments for 5 days before fixation and staining. Briefly, cells/plates were washed twice with ice cold PBS, fixed for 10 min with ice cold 100% methanol for 10 min, stained with a solution of 0.5% crystal violet in PBS with 25% methanol for 10 min at room temperature, followed by a final rinse in DI water and allowed to air dry.

## Results

### Immunohistochemical analysis of BMI1 expression in human and canine OSA tissues

**Fig 1:**
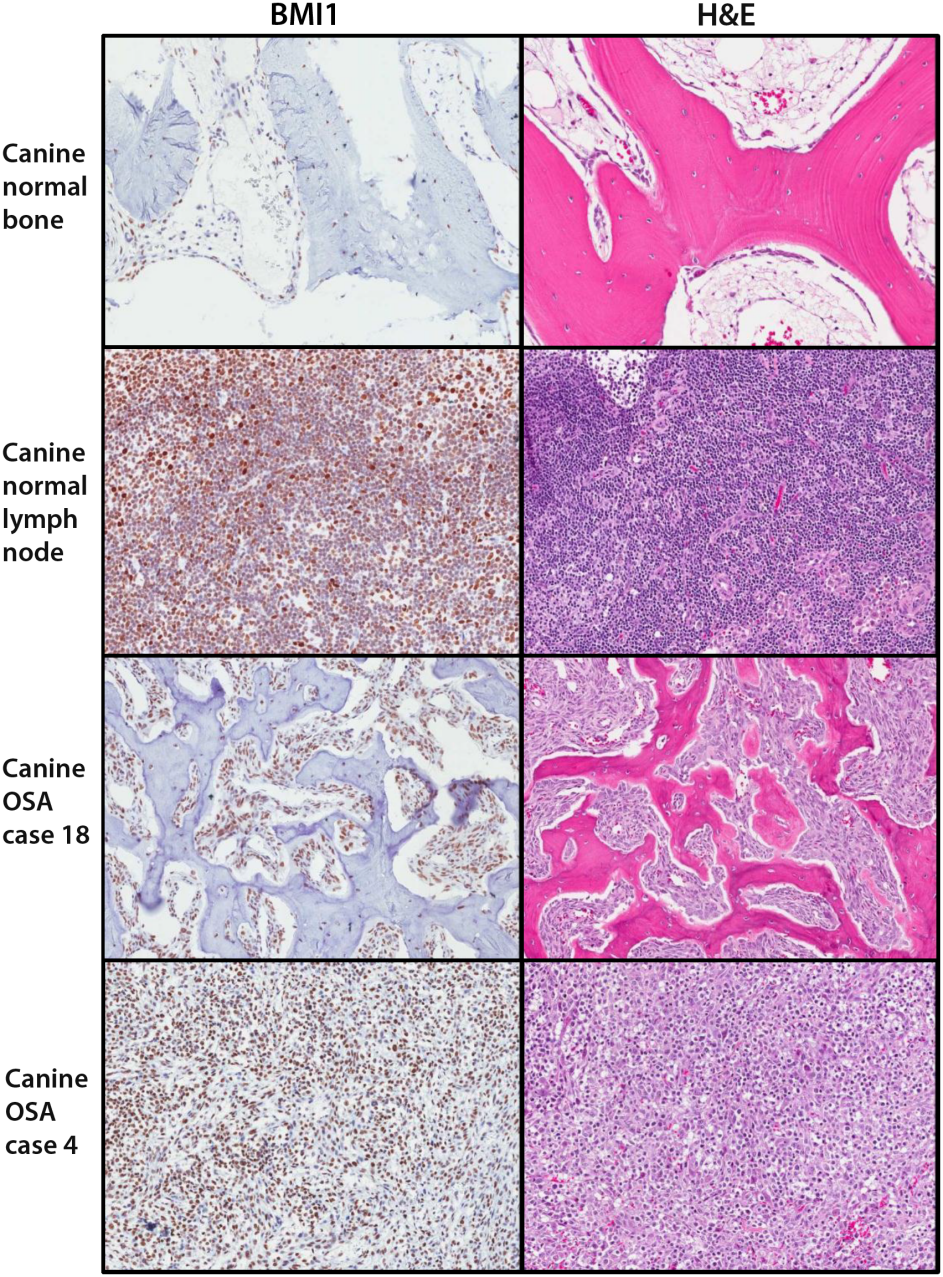
IHC analysis of BMI1 expression in canine lymph node, OSA, and adjacent normal bone. Representative images of BMI1 expression and corresponding H&E staining in canine normal adjacent bone, normal lymph node, and two canine OSA tissues corresponding to cases 18 and 4 from Table 1. Nuclear staining was visualized in adjacent normal bone. Canine lymph node demonstrated variable nuclear staining of individual lymphocytes and served as a positive control. BMI1 staining of canine OSA tissues was also localized to the nucleus and varied in intensity.

BMI1 expression was initially analyzed in 31 primary and 7 metastatic OSA samples from 33 canine patients (Table 1). Canine patients included 12 males and 21 females, with median age of 9. In this sample set, the radius was the most common site for primary canine OSA, while the lung was the most common metastatic site. Immunohistochemical staining revealed BMI1 expression in all 31 primary canine OSA samples and 6 out of 7 metastatic samples. For all BMI1 positive canine samples, staining was observed in at least 85% of the cells within the tumor sample (Fig 1 and Table 1). Normal canine lymph node served as a positive control.

**Table 1:** Semiquantitative expression analysis of BMI1 in canine primary and metastatic OSA.

Case #	Age	Breed	Gender	Site Location (primary, met)	Diagnosis	BMI1% (primary, met)
1	10	Mix	MC	Proximal humerus	Osteosarcoma	90
2	10	Rott	FS	Distal radius	Osteoblastic Osteosarcoma	80
3	11	Lab	MC	Proximal humerus	Osteoblastic Osteosarcoma	100
4	9	Grt Dane	FS	Distal radius, Lung	Chondroblastic Osteosarcoma	100, 100
5	7	Eng Mast	FS	Distal radius	Osteosarcoma	100
6	5	Anatolian Sh	MC	Distal radius	Osteoblastic Osteosarcoma	100
7	10	Greyhound	MC	Proximal humerus	Osteoblastic Osteosarcoma	100
8	11	Germ Sh.	FS	Proximal radius	Osteosarcoma	100
9	9	Lab	FS	Proximal humerus	Telangiectatic Osteosarcoma	100
10	10	Rott	FS	Proximal tibia	Osteosarcoma	100
11	11	GSH Pointer	MC	Proximal humerus	Chondroblastic Osteosarcoma	90
12	9	Lab	FS	Distal femur	Osteoblastic Osteosarcoma	100
13	7	Leonberger	FS	Distal radius, Lung	Osteosarcoma	90, NEG
14	9	Rott	FS	Distal femur	Osteosarcoma	100
15	5	Rhod Ridge	M	Distal radius	Osteosarcoma, Mixed type	100
16	7	Gold Ret	FS	Distal femur	Osteosarcoma	100
17	9	Gold Ret	MC	Mid ulna	Osteosarcoma	90
18	9	Rott	MC	Distal tibia	Osteosarcoma	100
19	7	Mix	FS	Distal femur	Osteoblastic Osteosarcoma	100
20	11	Curly C Ret	FS	Proximal humerus	Osteoblastic Osteosarcoma	90
21	8	Mix	FS	Distal tibia	Osteosarcoma Giant cell type	100
22	7	Rott	FS	Distal femur	Osteosarcoma	100
23	10	Mix	MC	Proximal tibia, Lung	Chondroblastic Osteosarcoma	100, 80
24	7	Gr Pyr	FS	Distal tibia, Lung	Osteosarcoma	100, 100
25	5	Bernese MD	MC	Distal radius, Lung	Osteosarcoma	90, 100
26	11	Germ Sh.	FS	Distal tibia	Osteosarcoma	90
27	6	Rott	FS	Proximal tibia	Fibroblastic Osteosarcoma	90
28	7	Malamute	FS	Distal radius	Osteosarcoma	90
29	8	St. Bern	FS	Distal tibia	Osteosarcoma	90
30	7	Mix	FS	Distal radius	Osteosarcoma	90
31	2	Mix	MC	Distal radius	Fibroblastic Osteosarcoma	90
32	6	Old English	FS	Lung	Metastatic Osteosarcoma	100
33	9	Mix	MC	Lung	Metastatic Osteosarcoma	90

The relative percentage of BMI1 expressing cells was measured in OSA samples derived from 33 canine patients. Following IHC with the anti-BMI1 antibody, cells with visible nuclear staining were scored as positive. MC = male castrated, FS = female spayed, NEG = no staining detected.

**Fig 2:**
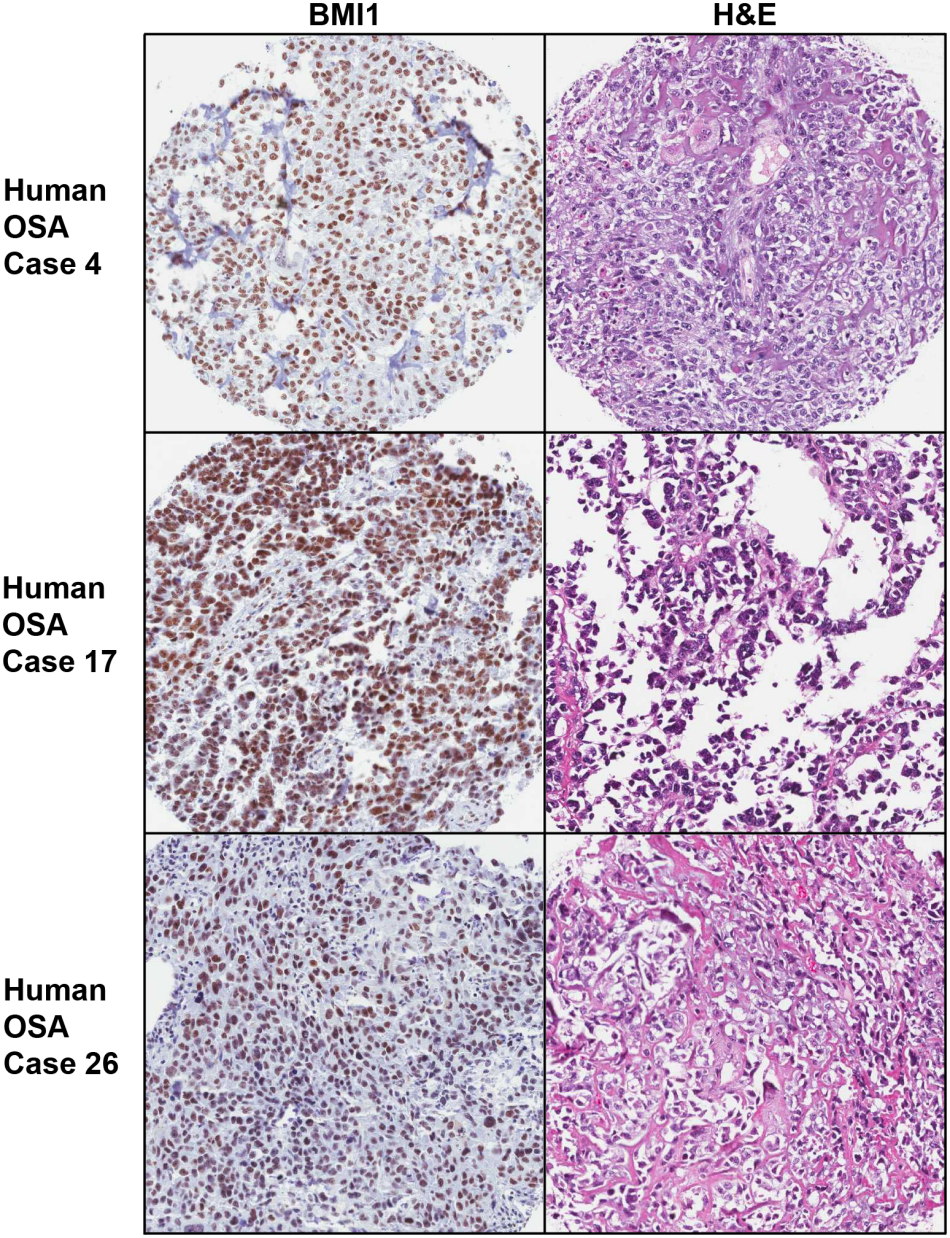
IHC analysis of BMI1 expression in human OSA. Representative images of BMI1 expression and corresponding H&E staining in human OSA tissue microarray samples corresponding to cases 4, 17, and 26 from Table 2. Similar to canine tissues, BMI1 staining of human OSA tissues was intense and localized to the nucleus.

BMI expression was determined via immunohistochemistry in a human OSA tissue microarray containing both primary and metastatic OSA tissues. Human samples included 17 primary and 10 metastatic OSA samples from 27 patients (Table 2). The patients included 18 males and 9 females with median age of 13. In this sample set, the femur was the most common site for primary OSAs, while the lung was the most common metastatic site. Immunohistochemical staining revealed BMI1 expression in 14 out of 17 human primary OSA samples and 10 out of 10 metastatic samples. Among those, 14 primary and 8 metastatic samples showed varying degrees of nuclear staining in 100% of the cells within the sample (Fig 2 and Table 2), while 2 metastatic samples revealed similar staining in about 50% of observed cells (data not shown).

**Table 2:** Semiquantitative expression analysis of BMI1 in human primary and metastatic OSA.

Case #	Age	Gender	Site Location	Diagnosis	BMI1%
1	80	M	Hip	Osteosarcoma	100
2	13	F	Femur	Osteosarcoma	100
3	13	F	Humerus	Osteogenic Osteosarcoma	100
4	55	M	Femur	Osteosarcoma	100
5	13	F	Right Distal Femur	Osteoblastic Osteosarcoma	100
6	62	M	Left Femur	Osteosarcoma	100
7	13	M	Left Ulna	Osteosarcoma	NEG
8	13	M	Left forearm	Osteosarcoma	100
9	14	M	Left Tibia	Osteosarcoma	100
10	13	F	Right Distal Femur	Osteosarcoma	100
11	21	M	S1 Nerve Root	Chondroblastic Osteosarcoma	100
12	21	M	Right Distal Femur	Osteosarcoma	100
13	37	M	Tibia	Chondroblastic Osteosarcoma	NEG
14	59	F	Hip	Chrondroblastic Osteosarcoma	100
15	26	M	Femur	Osteosarcoma	NEG
16	11	F	Femur	Osteosarcoma	100
17	11	F	Femur	Osteosarcoma	100
18	11	F	Lung	Metastatic Osteosarcoma	100
19	23	M	Lung	Metastatic Osteosarcoma	100
20	22	M	Lung	Metastatic Osteosarcoma	100
21	14	M	Left Upper Lung	Metastatic Osteosarcoma	100
22	11	M	Right Chest Mass	Metastatic Osteosarcoma	100
23	10	M	Right Lower Lobe Lung	Metastatic Osteosarcoma	100
24	10	M	Right Lower Lobe Lung	Metastatic Osteosarcoma	50
25	21	M	Left Lung	Metastatic Osteosarcoma	50
26	65	M	Left Lower Lung Lobe	Metastatic Osteosarcoma	100
27	11	F	Left Pleural Lining	Metastatic Osteosarcoma	100

The relative percentage of BMI1 expressing cells was measured in human OSA samples Following IHC with the anti-BMI1 antibody, cells with visible nuclear staining were scored as positive. NEG = no staining detected.

### BMI1 protein expression is upregulated in human and canine OSA cell lines

**Fig 3:**
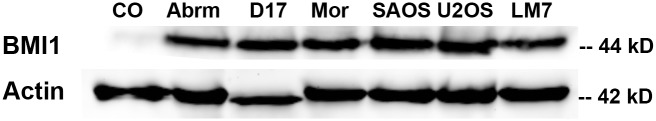
Protein expression of BMI1 in human and canine OSA cell lines. Western blot analysis of canine and human OSA cell lines revealed high expression of BMI1 relative to the normal canine osteoblasts (CO). Due to close proximity and strong signals for both BMI1 and Actin, two identical gels were loaded and protein electrophoresis and transfer were performed in parallel. Separate membranes were then probed with antibodies against BMI1 and Actin as shown. Abrm = Abrams, Mor = Moresco.

BMI1 protein expression was determined by western blot analysis for 3 human (SAOS-2, U2OS, and LM7) and 3 canine (Abrams, D17, Moresco) OSA cell lines and 1 normal canine osteoblast cell line. All OSA cell lines showed relatively high levels of BMI1 protein as compared to the control canine osteoblast cell line, where only a very weak band was observed (Fig 3).

### siRNA-mediated BMI1 Knockdown (KD) results in decreased BMI1 RNA and protein expression in canine OSA cells

**Fig 4:**
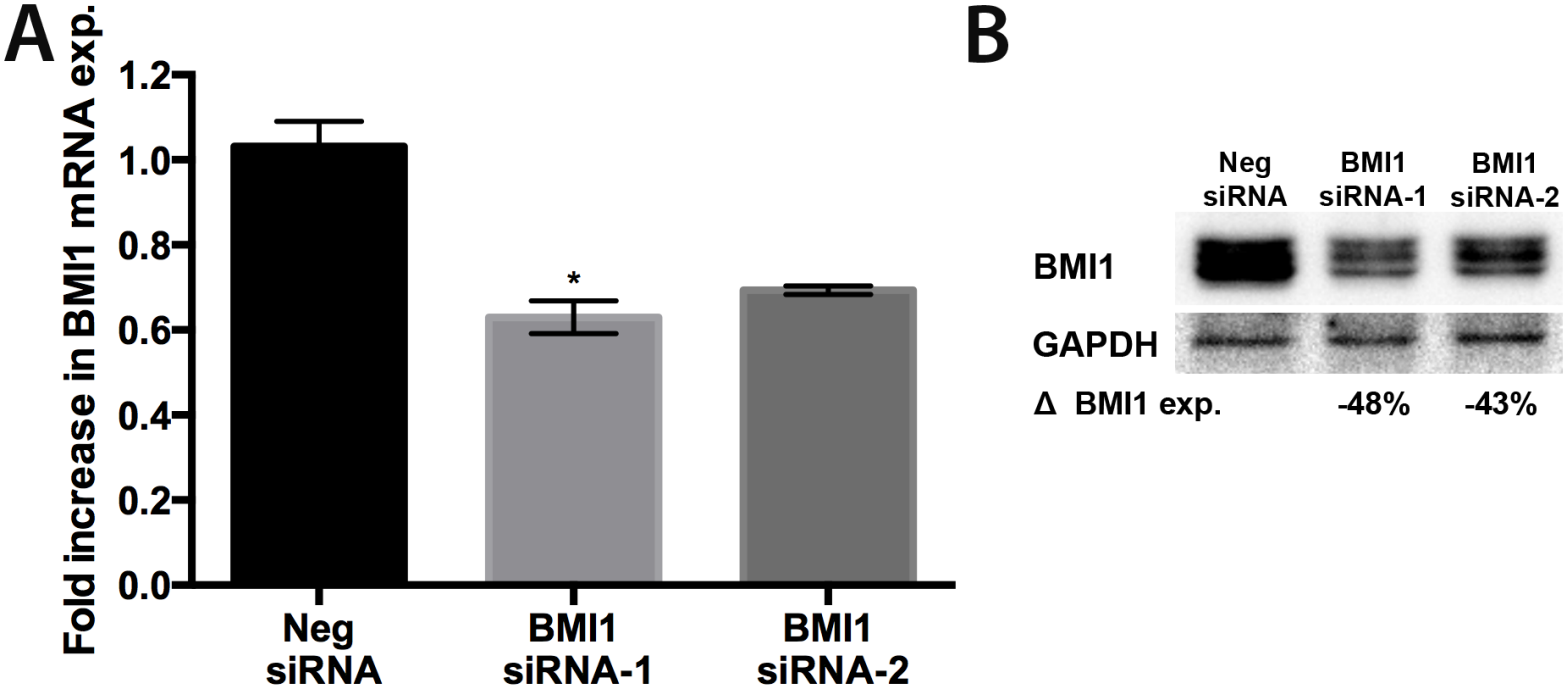
Expression of BMI1 mRNA and protein following siRNA knockdown in canine Abrams OSA cells. (A) Fold increase in mRNA expression in Neg siRNA, BMI1 siRNA-1, and BMI1 siRNA-2 transfected samples relative to untreated samples. Compared to the negative control siRNA, both BMI1 siRNA-1 and siRNA-2 show a trend for decreased BMI1 mRNA expression, although only the BMI1 siRNA-1 treated samples showed statistical significance. Error bars represent SD and statistical analysis was performed using Kruskal-Wallis with Dunn’s multiple comparisons test. *p<0.05 (B) Similarly, Western Blot Analysis confirmed that both siRNA-1 and siRNA-2 transfected cells expressed lower levels of BMI1 protein when compared to the Neg siRNA treated cells. BMI1 band densities were calculated, normalized to GAPDH, and displayed as a percent change in expression relative to Neg siRNA control.

The Abrams OSA cell line was transfected with canine BMI1 siRNAs and relative expression of BMI1 RNA and protein was assessed 72hrs later. Transfection with canine BMI1 siRNA-1 and 2 resulted in a 37% and 31% decrease in *BMI1* RNA expression, respectively (Fig 4A). A statistically significant decrease in RNA expression was observed with siRNA-1 (p<0.05). Western blot assay revealed similar decreases in BMI1 protein expression when Abrams cells were treated with BMI1 siRNA-1 (48%) or 2 (43%) as compared to negative control siRNA (Fig 4B).

### siRNA-mediated BMI1 Knockdown (KD) results in decreased cell viability in canine OSA cells

**Fig 5:**
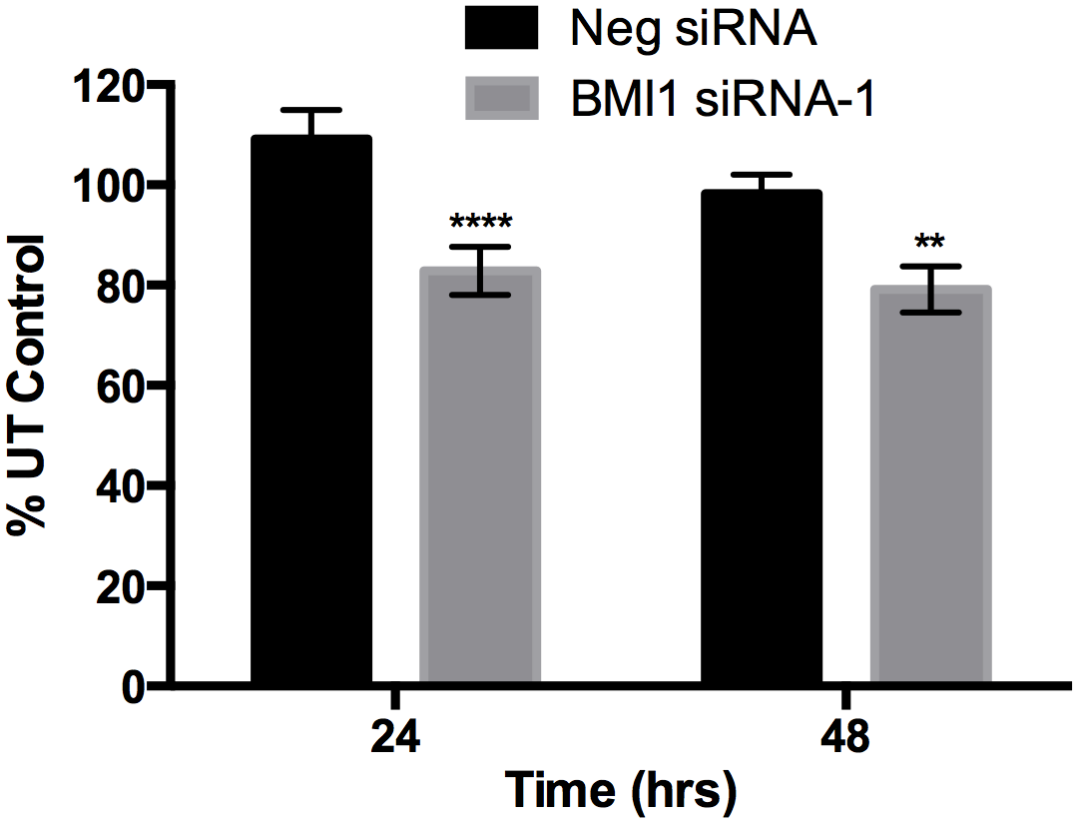
Effect of BMI1 siRNA transfection on cell viability in Abrams OSA cells. MTS assay showing decreased cell viability in Abrams canine OSA cells 24 and 48hrs after transfection with Neg siRNA and BMI1 siRNA-1. Absorbance data is shown as a percent of untreated (UT) control cells. Statistical analysis was performed using student’s t-test. ** p<0.01, **** p<0.0001.

To test the effect of BMI1 KD on OSA cell viability, the Abrams cell line was transfected with the canine BMI1 siRNA-1 and cell viability was measured using an MTS assay 24 and 48hrs later. Compared to negative control siRNA, a statistically significant decrease in cell viability was observed at 24hrs (p<0.0001) and 48hrs (p<0.01) for those cells transfected with the BMI1 siRNA-1 (Fig 5).

### siRNA-mediated BMI1 knockdown sensitizes OSA cells to chemotherapy

**Fig 6:**
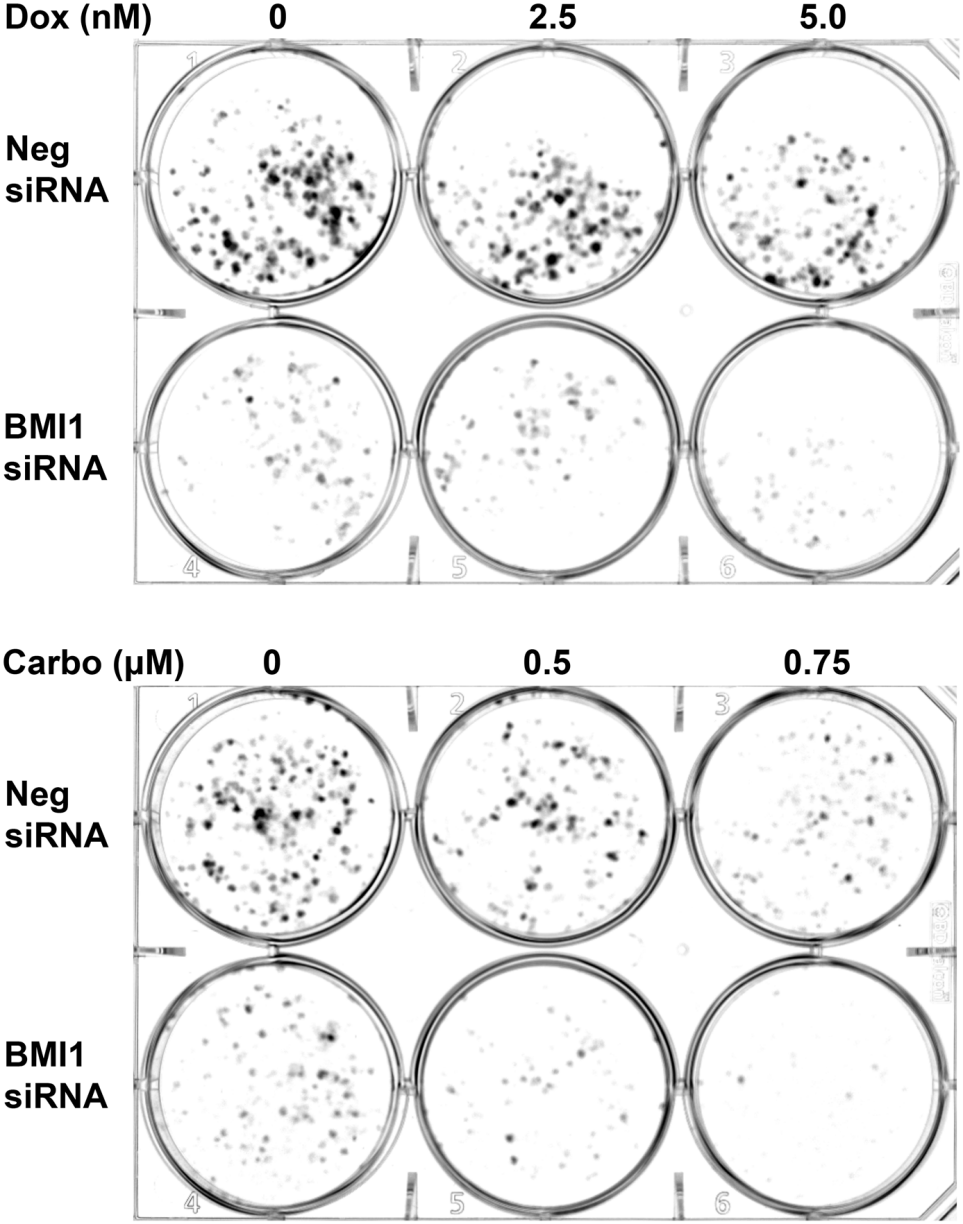
Effects of BMI1 siRNA transfection combined with chemotherapy on colony formation of Abrams OSA cells. BMI1 siRNA-1 transfection resulted in decreased colony formation when compared with Neg siRNA treated cells. The addition of Carboplatin (Carbo) or Doxorubicin (Dox) treatment to BMI1 siRNA-1 transfected Abrams cells resulted in decreased colony formation and growth compared to identically treated Negative siRNA transfected Abrams cells.

To test the influence of BMI1 knockdown on drug-mediated cell toxicity, negative siRNA and BMI1 siRNA-1 treated Abrams OSA cells were exposed to increasing doses of Carbo (0, 0.5, and 0.75μM) or Dox (0, 2.5, 5.0nM) and evaluated in a clonogenic assay. Fewer colonies were observed in wells where cells were pre-treated with BMI1 siRNA-1 compared to cells pre-treated with negative siRNA and a similar, dose dependent effect was observed for both carboplatin and doxorubicin (Fig 6). Pretreatment with BMI1 siRNA followed by carboplatin or doxorubicin treatment resulted in lower colony formation as compared to either treatment alone.

### PTC-209 decreases BMI1 and increases p16 protein expression in canine OSA cell lines

**Fig 7:**
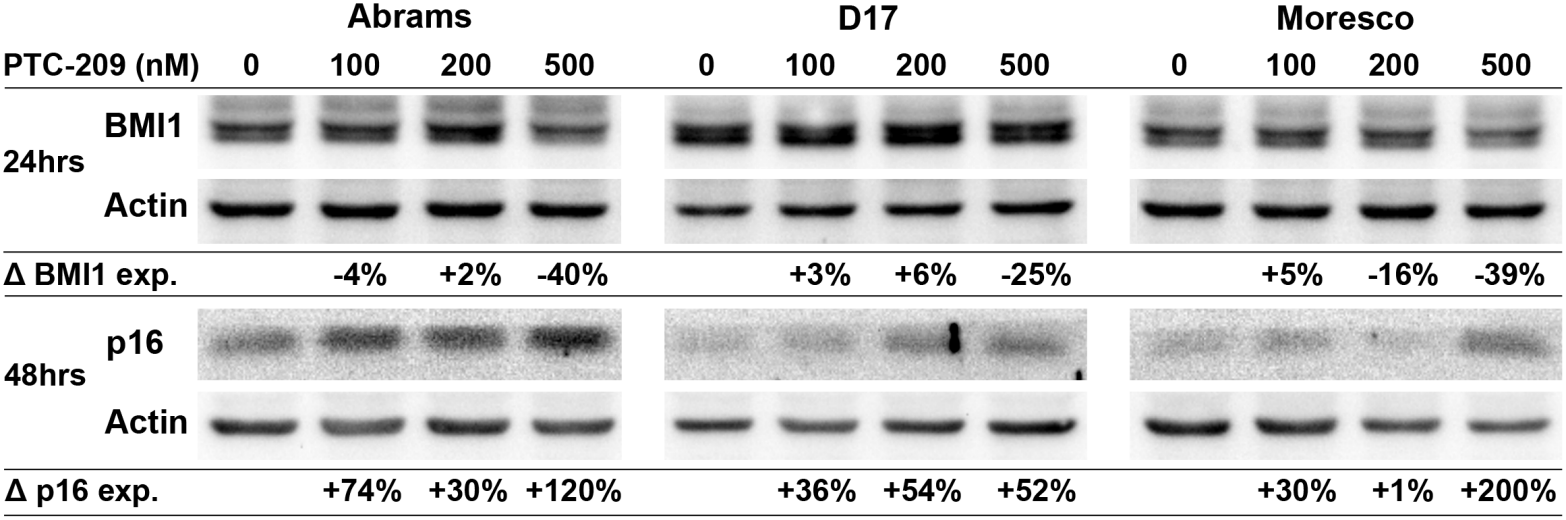
Effect of PTC-209 on BMI1 and p16 protein expression in canine OSA cell lines. Western blot showing decreased BMI1 protein expression and increased p16 protein expression in Abrams, D17, and Moresco canine OSA cell lines after 24hrs (BMI1) and 48hrs (p16) of PTC-209 treatment. Protein band densities were calculated for each sample, values were normalized to actin, and then shown as a percent change in expression relative to vehicle control.

Canine OSA cell lines were treated with PTC-209 (100, 200, or 500nM) for 24hrs and BMI1 expression was analyzed in a western blot assay (Fig 7). Compared to vehicle control, BMI1 protein expression decreased by 40% and 25% in the Abrams and D17 cell lines, respectively, following 500nM PTC-209 treatment. In the Moresco cell line, BMI1 protein expression decreased by 16% and 39% following 200nM and 500nM PTC-209 treatment, respectively, as compared to vehicle control. To assess changes in p16 protein expression, canine OSA cell lines were treated with similar doses of PTC-209 for 48hrs and analyzed with western blot (Fig 7). Increases in p16 protein levels could be observed in all cell lines beginning at 100nM PTC-209 and were highest at the 500nM PTC-209 dose for Abrams (120% increase) and Moresco (200% increase), but appeared to top out at 200nM for the D17 cell line (54% increase).

### BMI1 inhibitor PTC-209 decreases cell viability in canine OSA cell lines

**Fig 8:**
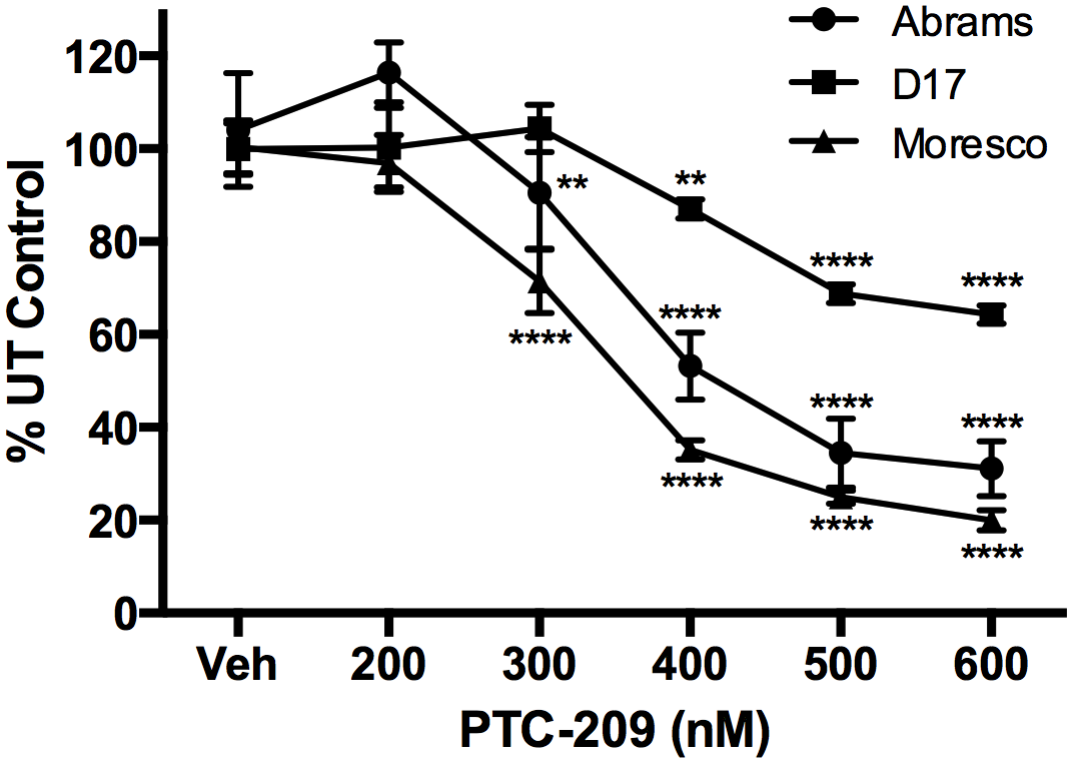
Effect of PTC-209 treatment on viability of canine OSA cell lines. MTT assay showing dose dependent decreases in cell viability following 72hrs of PTC-209 treatment. Absorbance data is shown as a percent of untreated (UT) control cells. Error bars reflect SD and statistical analysis was performed using 2-way ANOVA with Tukey’s multiple comparisons test. p-values reflect significance as compared to vehicle control.** p<0.01, **** p<0.0001.

An MTT assay was used to assess the viability of canine OSA cell lines following 72hrs of exposure to PTC-209. Significant decreases in cell viability were observed beginning at 300nM PTC-209 with the Abrams (P < 0.01) and Moresco (p < 0.001) cell lines and at 400nM PTC-209 with the D17 (p < 0.01) cell line (Fig 8). No significant differences were observed between groups treated with 500 and 600nM PTC-209, where cell viability decreased by 36%, 64%, and 80% for the D17, Abrams, and Moresco cell lines, respectively.

### Combination treatment with Dox or Carbo and BMI1 inhibitor PTC-209

**Fig 9:**
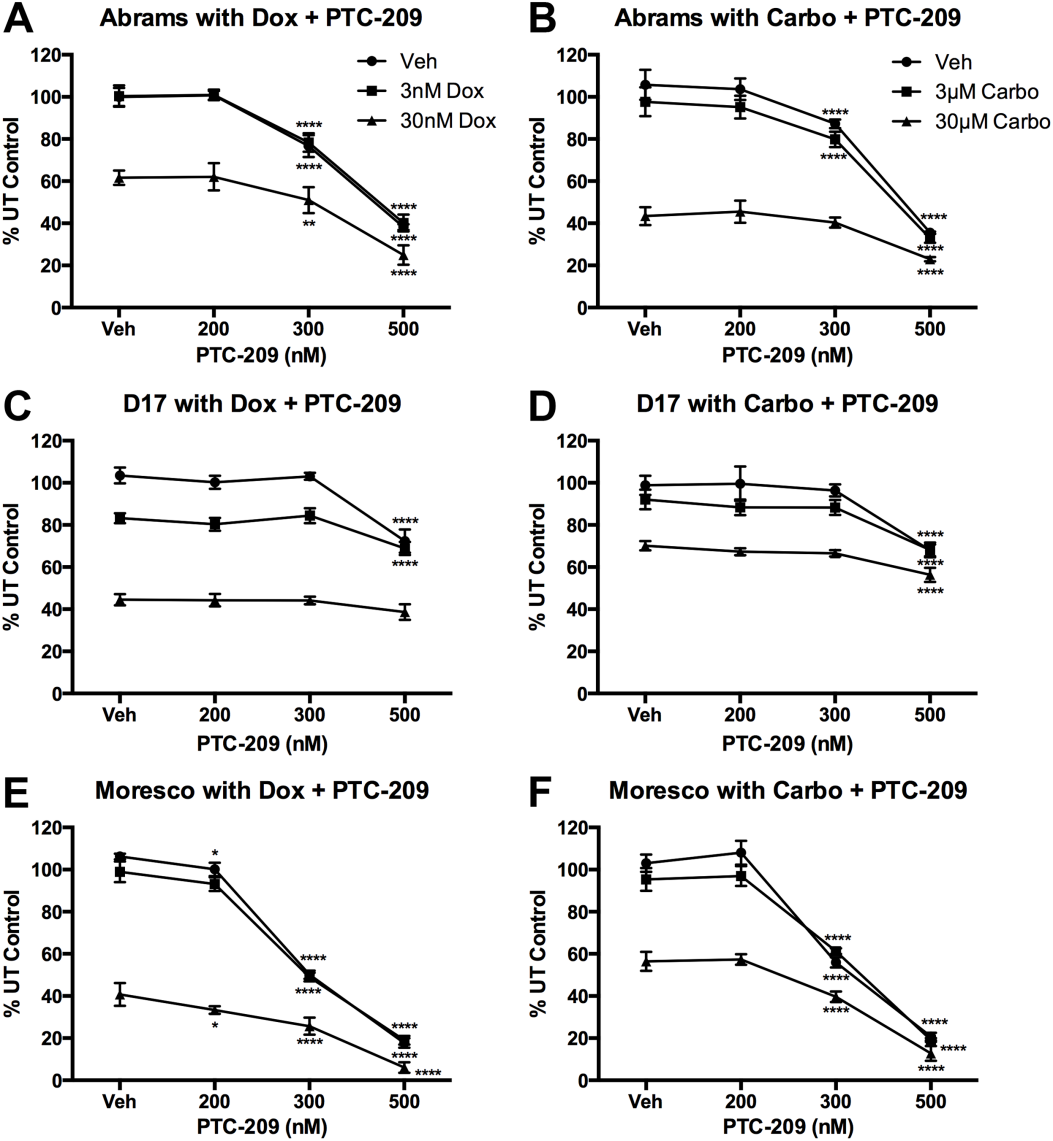
Effect of combined chemotherapy and PTC-209 treatment on viability of canine OSA cell lines. MTT assay following 72hrs of combined treatment with doxorubicin (Dox) or carboplatin (Carbo) with PTC-209. (A) Abrams treated with Dox and PTC-209. (B) Abrams treated with Carbo and PTC-209. (C) D17 treated with Dox and PTC-209. (D) D17 treated with Carbo and PTC-209. (E) Moresco treated with Dox and PTC-209. (F) Moresco treated with Carbo and PTC-209. Absorbance data is shown as a percent of untreated (UT) control cells. Error bars reflect SD and statistical analysis was performed using 2-way ANOVA with Tukey’s multiple comparisons test. p-values reflect significance as compared to PTC-209 vehicle control within each dose of Dox or Carbo. *p< 0.05, ** p<0.01, **** p<0.0001.

Canine OSA cell lines were exposed to varying doses of Dox or Carbo in combination with PTC-209 and cell viability was measured using an MTT assay. None of the cell lines treated with the lower doses of Dox (3nM) or Carbo (3μM) in combination with PTC-209 showed a significant decrease in cell viability as compared to dose matched single treatment PTC-209 (Fig 9). Differences observed between the D17 groups treated with 3nM Dox + PTC-209 (veh, 200, 300nM) and dose matched single treatment PTC-209 are attributed solely to the low dose Dox treatment and no difference was observed between the two groups when exposed to 500nM PTC-209 (Fig 9C).

When the Abrams cell line was treated with PTC-209 in combination with 30nM Dox, a significant decrease in cell viability was observed at 300 and 500nM PTC-209, compared to Dox alone, which were also significantly different from matched single treatment PTC-209 at 300nM (p < 0.0001) and 500nM (p < 0.001), respectively (Fig 9A). Likewise, when combined with 30μM Carbo, a significant decrease in cell viability was observed at 300 and 500nM PTC-209, compared to Carbo alone, which were also significantly different from their matched single treatment PTC-209 at 300nM (p < 0.0001) and 500nM (p < 0.001), respectively (Fig 9B).

Similarly, when the Moresco cell line was treated with PTC-209 in combination with 30nM Dox, a significant decrease in cell viability was observed at 200, 300, and 500nM PTC-209, compared to Dox alone, which were also significantly different from their matched single treatment PTC-209 group (p < 0.0001 for all, Fig 9E). Likewise, when combined with 30μM Carbo, a significant decrease in cell viability was observed at 300 and 500nM PTC-209, compared to Carbo alone, which were also significantly different from their matched single treatment PTC-209 at 300nM (p < 0.0001) and 500nM (p < 0.01), respectively (Fig 9F).

When the D17 cell line was treated with PTC-209 in combination with 30nM Dox, no significant decrease in cell viability was observed at any dose of PTC-209, as compared to Dox alone (Fig 9C). When combined with 30μM Carbo, however, a significant decrease was observed at 500nM PTC-209, compared to Carbo alone, which was also significantly different from matched single treatment PTC-209 at 500nM (p < 0.001, Fig 9D).

## Discussion

BMI1 has recently been shown to be upregulated in a variety of human cancers, including brain, breast, prostate, colon, and OSA [8, 20]. To our knowledge, only one study examining lymphoma cells has evaluated BMI1 expression in canine tumors [21]. Here we report that BMI1 is highly expressed in canine OSA, similar to what has been reported in human OSA tissues [8, 10]. In addition, we report for the first time that elevated BMI1 expression is maintained in both human and canine OSA metastases. Furthermore, canine OSA cell lines were found to express high levels of BMI1 protein, and siRNA mediated BMI1 knockdown or small molecule BMI1 inhibition in canine OSA cells results in a reduction in cellular proliferation and an increased sensitivity to the chemotherapeutic drugs carboplatin and doxorubicin.

We report positive BMI1 expression in 82% (14/17) human osteosarcoma samples analyzed, which is higher than the 56% reported previously [8]. Although it is possible that these differences represent real variability of BMI1 expression between sample sets, it is also possible that this discrepancy in reported expression results from the dissimilar BMI1 antibodies and specific criterion used to determine positive BMI1 expression. Of note are distinct differences in IHC staining patterns observed between these 2 studies. In the previous report, BMI1 IHC staining was observed as being both nuclear and cytoplasmic, while the BMI1 staining observed in our study was almost exclusively nuclear. This is important because the presence of cytoplasmic staining observed in the previous report was a defining factor for scoring a sample as positive for BMI1 expression. While small, a different study previously reported that 3 out of 3 human OSA samples expressed BMI1 protein that was exclusively localized in the nucleus of OSA cells [10]. What is unknown, however, is whether the specific localization of BMI1 as cytoplasmic versus nuclear significantly influences the development or progression of OSA as has been shown in other cancers [22].

The small molecule BMI1 inhibitor PTC-209 has been shown to decrease BMI protein expression and induce cytotoxicity in human colorectal cancer cells, but its effects on canine cancer cells or OSA cells has not previously been evaluated. We observed a decrease in BMI1 protein expression in all three canine OSA cell lines following treatment of PTC-209 at 500nM, which is consistent with previous reports using human tumor cell lines [7]. Furthermore, it has known that BMI1 negatively regulates expression of p16/INK4A [6], and as such, we observed an increase in p16 protein expression in all three canine OSA cell lines following PTC-209 inhibition of BMI1. Others have shown that p16 expression predicts the clinical response to chemotherapy in human OSA patients [23], which is in alignment with our findings and lends support to the notion that targeting BMI-1 may enhance response to chemotherapy in clinical OSA patients.

We found that siRNA-mediated knockdown of BMI1 in Abrams cells and BMI1 inhibition by PTC-209 in all three canine OSA cell lines (Abrams, D17, Moresco) decreased cell proliferation *in vitro* based on colorimetric viability assays. While these findings agree with studies examining human SAOS-2 OSA cells [8], no such growth inhibition was attributed solely to BMI1 expression knockdown in the human OSA cell lines 143B or HOS [10]. It is possible that the methods used, specifically the use of knockdown models, may have given discordant results, or perhaps that contribution of BMI1 to cellular proliferation may be variable between cell lines. Indeed, we did observe significant differences in response to BMI1 inhibition between the three canine OSA cell lines, with respect to both BMI1 protein expression changes and cytotoxicity. The cell line that appeared to be the most resistant to the cytotoxic effects of BMI1 inhibition (D17, Fig 8), also showed the smallest decrease in BMI1 protein expression following treatment (Fig 7). Conversely, the cell line that showed the least resistance to the cytotoxic effects of BMI1 inhibition (Moresco, Fig 8), also showed the largest decrease in BMI1 protein expression following treatment (Fig 7). D17 also appeared to express the highest level of baseline BMI1 protein, compared to Abrams and Moresco, which may have contributed to its resistance to PTC-209 (Figs 3 and 7).

All three canine OSA cell lines showed significantly decreased cell viability following combination PTC-209 treatment and Dox or Carbo, except for D17 which showed significant differences with PTC-209 combined with Carbo, but not Dox. Our finding that BMI1 appears to contribute to chemoresistance in canine OSA cells is consistent with previous studies examining human OSA [8, 9] and other tumor cell lines [24, 25]. We specifically chose to evaluate the role of BMI1 in chemoresistance to carboplatin and doxorubicin as they represent standard of care in the management of canine OSA [26, 27].

It has long been known that tumors consist of heterogeneous cell populations in regard to growth and metastasis [28, 29]. Several studies have suggested that within the heterogenous tumor cell population, a subset of OSA cells with stem cell characteristics (cancer stem cells or CSCs) may be responsible for tumor growth and treatment resistance [30, 31]. A recent seminal study by Tomasetti and Vogelstein [32] supports the role of stem cell involvement in cancer showing a direct positive correlation between the number of stem cell divisions in the lifetime of a given tissue and the lifetime risk of cancer developing in that tissue. Although evidence exists that BMI1 is involved in CSC self-renewal and tumorigenesis in other malignancies [20], it has not been specifically evaluated in putative OSA CSCs. While BMI1 has not been specifically evaluated as a putative CSC marker in OSA, our findings that BMI1 is highly expressed in metastases and contributes to chemotherapy resistance may implicate an important role for BMI1 in OSA CSCs. As such, additional studies aimed at evaluating the potential CSC properties of BMI1 positive OSA subpopulations may be warranted.

In conclusion, we demonstrate that BMI1 is highly expressed in canine and human primary and metastatic OSA and that, similar to what has been reported in human OSA cells, selective downregulation of BMI1 can sensitize canine OSA cells to the effects of chemotherapy. These results implicate a role for BMI1 in putative OSA CSCs and establish yet another similarity between the human and canine disease. Finally, these results demonstrate that targeting of BMI1 in OSA metastases may represent one approach to improve response to standard of care cytotoxic chemotherapy.

## References

[1] Sauvageau M, Sauvageau G. Polycomb group proteins: multi-faceted regulators of somatic stem cells and cancer. Cell stem cell. 2010;7(3):299–313. Epub 2010/09/02. 10.1016/j.stem.2010.08.002 . 20804967 PMC4959883

[2] Vissers JH, van Lohuizen M, Citterio E. The emerging role of Polycomb repressors in the response to DNA damage. Journal of cell science. 2012;125(Pt 17):3939–48. Epub 2012/10/30. 10.1242/jcs.107375 . 23104738

[3] van Lohuizen M, Verbeek S, Scheijen B, Wientjens E, van der Gulden H, Berns A. Identification of cooperating oncogenes in E mu-myc transgenic mice by provirus tagging. Cell. 1991;65(5):737–52. Epub 1991/05/31. . 1904008 10.1016/0092-8674(91)90382-9

[4] Raaphorst FM. Self-renewal of hematopoietic and leukemic stem cells: a central role for the Polycomb-group gene Bmi-1. Trends in immunology. 2003;24(10):522–4. Epub 2003/10/14. . 14552834 10.1016/s1471-4906(03)00241-2

[5] Molofsky AV, He S, Bydon M, Morrison SJ, Pardal R. Bmi-1 promotes neural stem cell self-renewal and neural development but not mouse growth and survival by repressing the p16Ink4a and p19Arf senescence pathways. Genes & development. 2005;19(12):1432–7. 10.1101/gad.1299505 15964994 PMC1151659

[6] Jacobs JJ, Kieboom K, Marino S, DePinho RA, van Lohuizen M. The oncogene and Polycomb-group gene bmi-1 regulates cell proliferation and senescence through the ink4a locus. Nature. 1999;397(6715):164–8. 10.1038/16476 . 9923679

[7] Kreso A, van Galen P, Pedley NM, Lima-Fernandes E, Frelin C, Davis T, et al Self-renewal as a therapeutic target in human colorectal cancer. Nature medicine. 2014;20(1):29–36. 10.1038/nm.3418 . 24292392

[8] Wu Z, Min L, Chen D, Hao D, Duan Y, Qiu G, et al Overexpression of BMI-1 promotes cell growth and resistance to cisplatin treatment in osteosarcoma. PLoS One. 2011;6(2):e14648 Epub 2011/02/12. 10.1371/journal.pone.0014648 21311599 PMC3032734

[9] Xie X, Ye Z, Yang D, Tao H. Effects of combined c-myc and Bmi-1 siRNAs on the growth and chemosensitivity of MG-63 osteosarcoma cells. Molecular medicine reports. 2013;8(1):168–72. Epub 2013/05/21. 10.3892/mmr.2013.1484 . 23685757

[10] Sasaki H, Setoguchi T, Matsunoshita Y, Gao H, Hirotsu M, Komiya S. The knock-down of overexpressed EZH2 and BMI-1 does not prevent osteosarcoma growth. Oncology reports. 2010;23(3):677–84. . 20127006 10.3892/or_00000684

[11] Khanna C, London C, Vail D, Mazcko C, Hirschfeld S. Guiding the optimal translation of new cancer treatments from canine to human cancer patients. Clin Cancer Res. 2009;15(18):5671–7. Epub 2009/09/10. 1078-0432.CCR-09-0719 [pii] 10.1158/1078-0432.CCR-09-0719 19737961 PMC2748812

[12] Paoloni M, Davis S, Lana S, Withrow S, Sangiorgi L, Picci P, et al Canine tumor cross-species genomics uncovers targets linked to osteosarcoma progression. BMC genomics. 2009;10:625 Epub 2009/12/24. 10.1186/1471-2164-10-625 20028558 PMC2803201

[13] Fenger JM, London CA, Kisseberth WC. Canine osteosarcoma: a naturally occurring disease to inform pediatric oncology. ILAR journal / National Research Council, Institute of Laboratory Animal Resources. 2014;55(1):69–85. Epub 2014/06/18. 10.1093/ilar/ilu009 . 24936031

[14] Maeda J, Yurkon CR, Fujisawa H, Kaneko M, Genet SC, Roybal EJ, et al Genomic instability and telomere fusion of canine osteosarcoma cells. PloS one. 2012;7(8):e43355 Epub 2012/08/24. 10.1371/journal.pone.0043355 22916246 PMC3420908

[15] Legare ME, Bush J, Ashley AK, Kato T, Hanneman WH. Cellular and phenotypic characterization of canine osteosarcoma cell lines. Journal of Cancer. 2011;2:262–70. Epub 2011/05/10. 21552385 10.7150/jca.2.262 PMC3088864

[16] Seo K, Holt R, Jung YS, Rodriguez CO Jr., Chen X, Rebhun RB. Fluoroquinolone-mediated inhibition of cell growth, S-G2/M cell cycle arrest, and apoptosis in canine osteosarcoma cell lines. PloS one. 2012;7(8):e42960 10.1371/journal.pone.0042960 22927942 PMC3424257

[17] Shahi MH, Holt R, Rebhun RB. Blocking signaling at the level of GLI regulates downstream gene expression and inhibits proliferation of canine osteosarcoma cells. PloS one. 2014;9(5):e96593 10.1371/journal.pone.0096593 24810746 PMC4014515

[18] Duan X, Jia SF, Zhou Z, Langley RR, Bolontrade MF, Kleinerman ES. Association of alphavbeta3 integrin expression with the metastatic potential and migratory and chemotactic ability of human osteosarcoma cells. Clin Exp Metastasis. 2004;21(8):747–53. . 16035619 10.1007/s10585-005-0599-6

[19] Harju VT, Alitalo R, Andersson LC. Divergent in vitro effects of recombinant interferons on human osteosarcoma cells. Bone. 1990;11(4):247–51. . 2122912 10.1016/8756-3282(90)90077-c

[20] Cao L, Bombard J, Cintron K, Sheedy J, Weetall ML, Davis TW. BMI1 as a novel target for drug discovery in cancer. Journal of cellular biochemistry. 2011;112(10):2729–41. 10.1002/jcb.23234 . 21678481

[21] Kim MC, D'Costa S, Suter S, Kim Y. Evaluation of a side population of canine lymphoma cells using Hoechst 33342 dye. Journal of veterinary science. 2013;14(4):481–6. 23820219 10.4142/jvs.2013.14.4.481 PMC3885743

[22] Fan C, He L, Kapoor A, Rybak AP, De Melo J, Cutz JC, et al PTEN inhibits BMI1 function independently of its phosphatase activity. Molecular cancer. 2009;8:98 10.1186/1476-4598-8-98 19903340 PMC2777864

[23] Borys D, Canter RJ, Hoch B, Martinez SR, Tamurian RM, Murphy B, et al P16 expression predicts necrotic response among patients with osteosarcoma receiving neoadjuvant chemotherapy. Human pathology. 2012;43(11):1948–54. 10.1016/j.humpath.2012.02.003 . 22578565

[24] Siddique HR, Parray A, Tarapore RS, Wang L, Mukhtar H, Karnes RJ, et al BMI1 polycomb group protein acts as a master switch for growth and death of tumor cells: regulates TCF4-transcriptional factor-induced BCL2 signaling. PloS one. 2013;8(5):e60664 10.1371/journal.pone.0060664 23671559 PMC3645992

[25] Siddique HR, Saleem M. Role of BMI1, a stem cell factor, in cancer recurrence and chemoresistance: preclinical and clinical evidences. Stem cells. 2012;30(3):372–8. 10.1002/stem.1035 . 22252887

[26] Selmic LE, Burton JH, Thamm DH, Withrow SJ, Lana SE. Comparison of carboplatin and doxorubicin-based chemotherapy protocols in 470 dogs after amputation for treatment of appendicular osteosarcoma. J Vet Intern Med. 2014;28(2):554–63. 10.1111/jvim.12313 . 24512451 PMC4857984

[27] Skorupski KA, Uhl JM, Szivek A, Allstadt Frazier SD, Rebhun RB, Rodriguez CO Jr. Carboplatin versus alternating carboplatin and doxorubicin for the adjuvant treatment of canine appendicular osteosarcoma: a randomized, phase III trial. Veterinary and comparative oncology. 2013 10.1111/vco.12069 . 24118677 PMC5012431

[28] Fidler IJ, Kripke ML. Metastasis results from preexisting variant cells within a malignant tumor. Science. 1977;197(4306):893–5. . 887927 10.1126/science.887927

[29] Fidler IJ. Biological heterogeneity of cancer: implication to therapy. Human vaccines & immunotherapeutics. 2012;8(8):1141–2. 10.4161/hv.19643 22854675 PMC3551889

[30] Basu-Roy U, Basilico C, Mansukhani A. Perspectives on cancer stem cells in osteosarcoma. Cancer letters. 2013;338(1):158–67. 10.1016/j.canlet.2012.05.028 22659734 PMC3552024

[31] He H, Ni J, Huang J. Molecular mechanisms of chemoresistance in osteosarcoma (Review). Oncology letters. 2014;7(5):1352–62. 10.3892/ol.2014.1935 24765137 PMC3997672

[32] Tomasetti C, Vogelstein B. Cancer etiology. Variation in cancer risk among tissues can be explained by the number of stem cell divisions. Science. 2015;347(6217):78–81. 10.1126/science.1260825 . 25554788 PMC4446723

